# Coffee intake and hypertension in Korean adults: results from KNHANES 2012–2016

**DOI:** 10.1186/s40885-023-00239-4

**Published:** 2023-07-15

**Authors:** Surabhi Shah, In-Jeong Cho, Whanhee Lee, Wook Bum Pyun, Eunhee Ha

**Affiliations:** 1 Department of Environmental Medicine, Ewha Womans University College of Medicine, Seoul, Republic of Korea; 2 Department of Internal Medicine, Ewha Womans University College of Medicine, Seoul, Republic of Korea; 3 School of Biomedical Convergence Engineering, College of Information and Biomedical Engineering, Pusan National University, Yangsan, Republic of Korea

**Keywords:** Coffee, Hypertension, Caffeine

## Abstract

**Background:**

Coffee is the most popular and widely consumed drink in the world. Coffee consumption seems to have both benefits and risk with respect to hypertension; results from studies evaluating effect of frequency of coffee consumption on risk of hypertension are mixed and inconsistent. Hence, we investigated the association of coffee consumption and hypertension in Korean adults.

**Methods:**

Data from Korean National Health and Nutrition Examination Survey (KNHANES) 2012–2016 was obtained and 12,133 eligible participants were selected. The coffee consumption was attained using a food frequency questionnaire. Subsequently coffee intake was grouped into two categories: ≤2 and > 2 servings per day. Hypertension status was defined as systolic blood pressure ≥ 140 mmHg or diastolic blood pressure ≥ 90 mmHg, use of antihypertensive drug treatment, or both. Multivariable logistic regression analysis was used to examine the association of coffee consumption and hypertension.

**Results:**

Logistic regression analysis showed that consumption of more than two servings of coffee a day was inversely associated with hypertension with odds ratio (OR) 0.84 (95% confidence interval [CI], 0.73–0.99). Similar results were seen in the propensity score-matched analysis (OR, 0.83; 95% CI, 0.69–0.98). Adults having age more than median value (OR, 0.76; 95% CI, 0.65–0.89) and normal cholesterol (OR, 0.84; 95% CI, 0.70–0.99) had significantly inverse association with hypertension, when coffee consumption was more than two servings daily.

**Conclusions:**

More than two servings of coffee intake per day was inversely associated with hypertension as compared to consumption of ≤ 2 servings coffee per day.

**Supplementary Information:**

The online version contains supplementary material available at 10.1186/s40885-023-00239-4.

## Background

Hypertension is a common, powerful, and modifiable risk factor for cardiovascular disease worldwide [1]. The prevalence of hypertension in 2015 was 31.1% and was associated with 14% of all deaths worldwide [2]. In Korea, a nationwide study based on the Korean National Health Insurance data showed that 33% of the population aged 30 years or older have hypertension. The diagnosis of hypertension increased from 3 million in 2002 to 10.1 million in 2019 [3]. Hypertension was associated with attributable risk of 21% for ischemic heart disease and 21% of cerebrovascular disease [4], and it is identified as a leading contributor to loss of disability adjusted life years [5]. Hypertension can be prevented by modification of dietary and lifestyle factors [6].

In particular, coffee consumption is associated with hypertension. Coffee is reported as most frequently consumed foods by Koreans [7], and a recent study showed that around 50% of adults at least have two cups of coffee in a day [8]. Studies have examined beneficial effects of coffee consumption on hypertension and influence of other factors like health behaviors [6, 9, 10].

It is reported that coffee consumption is associated with circulatory diseases, but the directionality of the association has been mixed. A meta-regression analysis showed a positive association between coffee consumption and total cholesterol [11]. While another meta-analysis showed that individuals with highest coffee consumption were at a low risk of metabolic syndrome [12]. Coffee has gained interest with regards to risks and benefits on cardiovascular system [13]. Coffee have been associated with blood pressure by antagonizing the adenosine receptors and having antihypertensive effects [14]. A meta-analysis reported that habitual coffee consumption was not associated with risk of hypertension [15], while another meta-analysis for observational studies presented inconstant results [16].

Results from previous review and meta-analysis studies regarding the association between coffee intake and hypertension are inconsistent. Hence, the aim of our study was to evaluate the association of coffee intake and hypertension in Korean adults using data from the Korean National Health and Nutrition Examination Survey (KNHANES).

## Methods

### Ethics statement

This study was approved by the Institutional Review Board of Ewha Womans University Seoul Hospital (No. SEUMC 2022-08-079). The requirement for written informed consent was waived due to the noninterventional, retrospective design of the study.

### Study population

The KNHANES is a nationally representative, cross-sectional survey conducted by the Korean Ministry of Health and Welfare. The KNHANES uses complex, stratified, multistage, probability cluster sampling method, which enables the collection of extensive and representative data concerning health and nutritional status in the noninstitutionalized civilian Korean population. Trained personnel collected data through health interviews, health examinations, and dietary interviews.

**Fig. 1:**
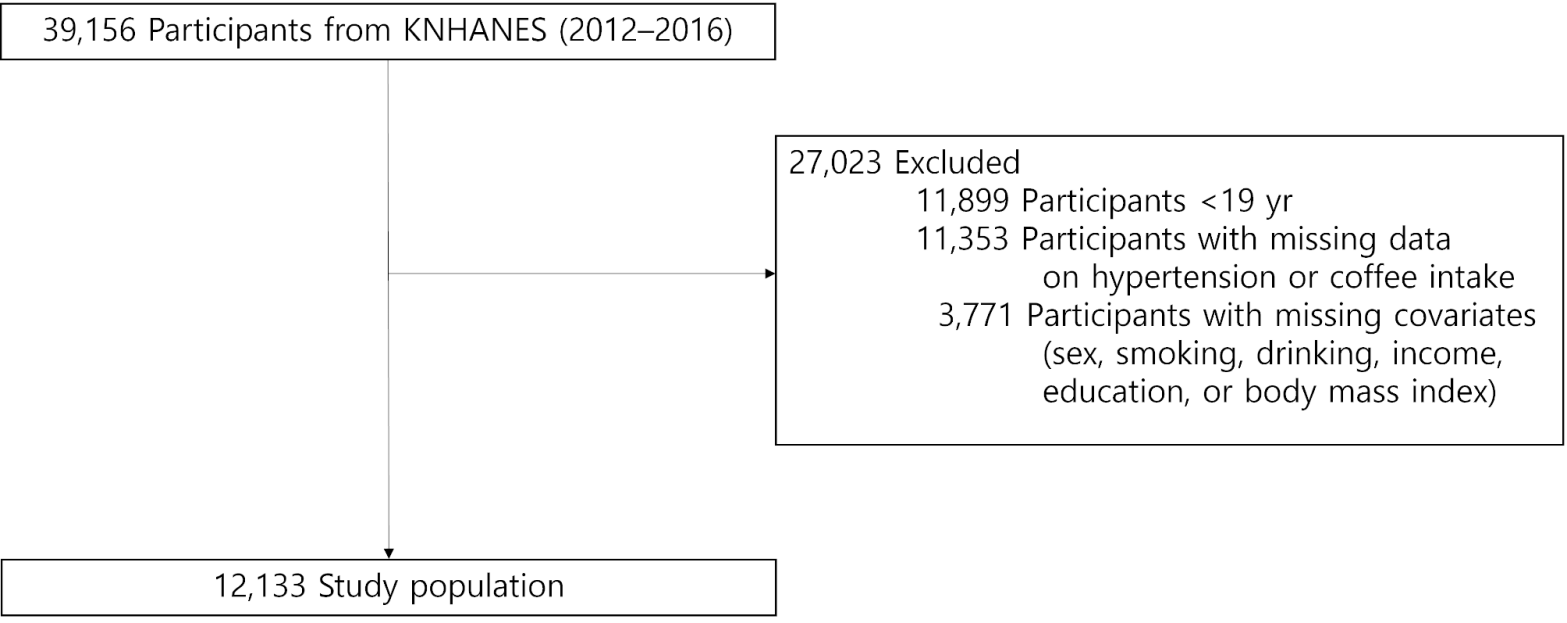
Selection of study participants from Korean National Health and Nutrition Examination Survey (KNHANES) 2012–2016

We analyzed data from KNHANES 2012–2016, which included 39,156 participants. Subjects were excluded from the study, if they were aged < 19 years (n = 11,899); had missing information on blood pressure, hypertension, and coffee consumption (n = 11,353); missing information on covariates sex, income, education, body mass index (BMI; n = 3,771). Finally, 12,133 participants were included in study (Fig. 1). When this survey was performed, written informed consent to use these data in further analyses was obtained. All the participants had the right to refuse to take part in the study, in accordance with the National Health Enhancement Act.

### Measurement and definitions of blood pressure and hypertension

In KNHANES, blood pressure (BP) was measured by four nurses in charge of BP measurement in the special investigation department of Korea Disease Control and Prevention Agency (KDCA) starting from phase IV (2007). The BP was not measured by automatic sphygmomanometer, but by a standard mercury sphygmomanometer (Baumanometer, WA Baum Co.). The BP was measured with participants in a sitting position after he or she had rested for at least 10 min. In all participants, the two BP measurement were taken at 5-minute intervals in a quiet atmosphere. The mean of these two measurements was used for the data analysis. The KDCA have been reinforcing quality control of BP measurement since 2011, by introducing a certification system, standardizing BP measurement and survey environment.

Hypertension was defined, based on the European Society of Hypertension (ESH) criteria [5]: systolic BP ≥ 140 mmHg, diastolic BP ≥ 90 mmHg, or use of antihypertensive medications. The BP was stratified into two categories: hypertension and no hypertension.

### Assessment of coffee consumption

Coffee consumption was measured with self-reported data obtained through food frequency questionnaire (FFQ). For each food item, there were nine categories from rarely to three times a day. In FFQ, the frequency of coffee intake was classified into the nine categories: rarely, one serving per month; two to three servings per month; one serving per week; two to three servings per week; four to six servings per week; one serving per day; two servings per day; and three or more servings per day. An updated semi-quantitative food frequency questionnaire (SQFFQ) used in the KNHANES from 2012 exhibited adequate reproducibility and modest validity [17]. Daily intake was calculated based on the midpoint of the assigned frequencies in each category for each food item. Coffee intake was calculated by multiplying the midpoints of frequencies by the number of servings consumed at any given time of intake. Frequency of coffee consumption was then classified into two groups (≤ 2 and > 2 servings per day) according to the mean daily coffee consumption of study participants.

### Covariates

Information regarding demographic and social factors was obtained during health interview using a standardized questionnaire. Heavy alcohol drinking was categorized as men drinking more than seven drinks and women drinking more than five drinks on average at one time for more than two times per week. Current smoking was defined as currently smoking with a smoking history of ≥ 100 cigarettes in a lifetime. Anthropometric measurements were obtained from well-trained staff following standard procedures across every phase of the KNHANES. Participants’ body weight and height were measured to the nearest 0.1 kg and 0.1 cm, respectively, while the participants wore light clothing without shoes. The BMI was calculated by dividing weight in kilograms to height in squared meters (kg/m^2^). BMI was divided into low weight group (< 18.5 kg/m^2^), normal weight group (18.5–25 kg/m^2^), and overweight group (> 25 kg/m^2^). After fasting for at least 8 h, blood samples were collected in the morning and analyzed at a central, certified laboratory. Plasma glucose and total cholesterol were measured using a Hitachi Automatic Analyzer 7600 (Hitachi). The diagnosis of diabetes was based on fasting plasma glucose (> 126 mg/dL), the current use of antidiabetic medication, or glycosylated hemoglobin ≥ 6.5%. Hypercholesterolemia was defined when total cholesterol was more > 240 mg/dL, diagnosed with hypercholesterolemia or taking medication [18]. Income level was categorized into quartiles and education level was categorized into two groups: high school and university graduate. Area of residence were classified into two groups as metropolitan (Seoul, Gyeonggi Province, Busan, Daegu, Incheon, Daejeon, and Ulsan) and non-metropolitan (included remaining areas).

### Statistical analysis

Data analysis was conducted in SAS ver. 9.4 (SAS Institute). Complex sampling design, as recommended by the KDCA was used during analysis. The characteristics of study participants is described in the form of frequency using chi-square test. Data on continuous variable was presented as mean and 95% confidence interval (CI). Due to difference in the baseline characteristics, propensity score matching was used to identify patients with similar baseline characteristics. The propensity score was estimated using non-parsimonious multivariable logistic regression model. Greedy nearest neighbor matching was performed. Standardized mean differences were estimated for all baseline characteristics. Association between coffee consumption and hypertension was assessed by logistic regression analysis, adjusting for age, sex, education, income, BMI, smoking, drinking, energy intake, diabetes diagnosis, and hypercholesterolemia diagnosis in all the participants and by sex. Interaction between coffee consumption and adjusting factor was evaluated. Further, subgroup logistic regression analysis was performed. We used SAS SURVEYLOGSTIC to account for the survey design and the complex sampling weights. All statistical tests were two-sided, and statistical significance was set at a P-value < 0.05.

## Results

The general characteristics of study participants according to their hypertension status are shown in Table 1. The mean age of participants who had hypertension was 49 years, 70% of the participants completed high school and 54% were overweight. 27% were smokers, 26% had hypercholesterolemia, and 17% had diabetes. The total daily calorie intake in hypertension participants was 2,328 kcal/day (Table 1). Distribution of coffee intake and hypertension by sex is shown in Table S1. 23% of men and 14% of women had hypertension. Around 32% men and 17% women had more than two servings of coffee per day (Table S1).

**Table 1:** General characteristics of study population

Characteristic	Total study population (n = 12,133)			After propensity-score matching (n = 5,172)		
	No hypertension (n = 9,774)	Hypertension (n = 2,359)	P-value	No hypertension (n = 4,004)	Hypertension (n = 1,168)	Standardized mean differences
Age (yrs)	38 (37–38)	49 (48–49)	< 0.001	41 (40–41)	48 (48–49)	0.01
Sex			< 0.001			–0.01
Man	3,900 (40)	1,403 (59)		2,146 (54)	841 (72)	
Woman	5,874 (60)	956 (41)		1,858 (46)	327 (28)	
Education level			< 0.001			–0.02
≤High school graduate	1,075 (11)	727 (30)		466 (12)	310 (27)	
≥College graduate	8,699 (89)	1,632 (70)		3,538 (88)	858 (73)	
Income (10,000 KRW/mo)			0.150			–0.02
< 100	2,131 (22)	580 (25)		888 (22)	272 (23)	
≥ 100–<200	2,469 (25)	581 (24)		1,025 (25)	287 (25)	
≥ 200–<300	2,510 (26)	611 (26)		1,030 (25)	319 (27)	
≥ 300	2,664 (27)	587 (25)		1,061 (25)	290 (25)	
Body mass index (kg/m^2^)			< 0.001			0.01
Low	513 (5)	16 (1)		167 (4)	7 (1)	
Normal	6,706 (69)	1,061 (45)		2,634 (66)	491 (42)	
Overweight	2,555 (26)	1,282 (54)		1,203 (30)	670 (57)	
Current smoker			< 0.001			–0.01
No	7,705 (78)	1,720 (73)		2,551 (64)	681 (58)	
Yes	2,069 (22)	639 (27)		1,453 (36)	487 (42)	
Heavy drinking			< 0.001			0.02
No	8,535 (87)	1,824 (77)		3,387 (85)	839 (72)	
Yes	1,239 (13)	535 (23)		617 (15)	329 (28)	
Total energy intake (kcal/day)	2,201 (2,175–2,226)	2,328 (2,276–2,381)	< 0.001	2,349 (2,309–2,388)	2,398 (2,398–2,561)	0.07
Diabetes mellitus			< 0.001			–0.03
No	9350 (96)	1,965 (83)		3,810 (95)	1,006 (86)	
Yes	424 (4)	394 (17)		194 (5)	162 (14)	
Hypercholesterolemia			< 0.001			–0.01
No	8,762 (90)	1,741 (74)		3,505 (86)	881 (75)	
Yes	1012 (10)	618 (26)		499 (14)	287 (25)	
Area of residence			0.270			–0.01
Metropolitan	6,313 (65)	1,470 (62)		2,483 (62)	714 (61)	
Non-metropolitan	3,461 (35)	889 (38)		1,521 (38)	454 (39)	

Data are presented as β (95% confidential interval) or number (%)

**Fig. 2:**
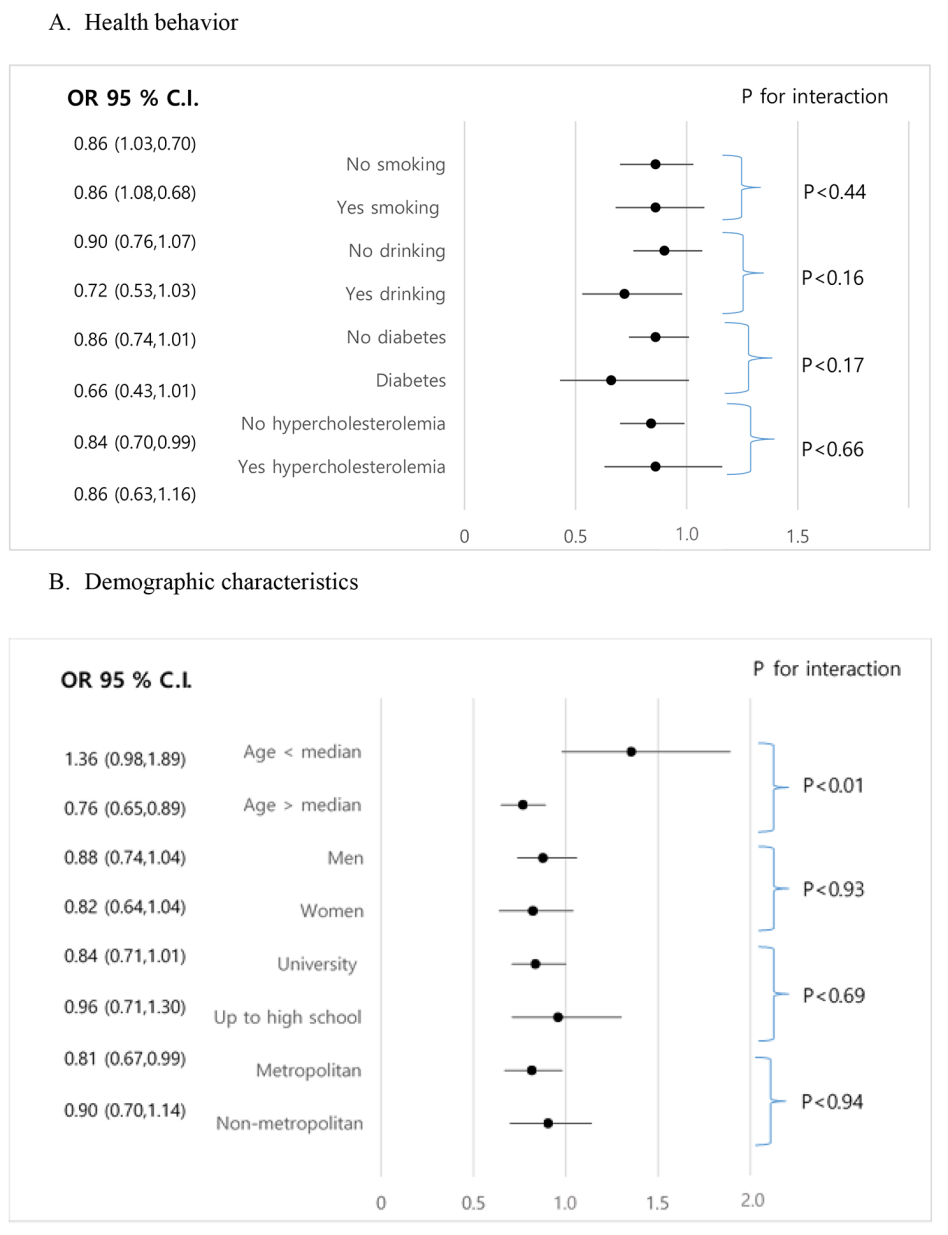
Subgroup analysis for association of daily coffee consumption with hypertension. Odds ratio (OR) was calculated by logistic regression analysis adjusted for age, sex, education, body mass index, current smoking, heavy drinking, diabetes, hypercholesterolemia, energy intake, income, and area of residence. **(A)** Stratified by health behavior. **(B)** Stratified by demographic characteristics. CI, confidence interval

In all study participants, consuming more than two servings of coffee intake in a day was associated with less odds with hypertension in total participants (odds ratio [OR], 0.85; 95% CI, 0.74–0.97) and propensity score-matched participants (OR, 0.83; 95% CI, 0.69–0.98), as compared to participants having up to two servings per day as shown in Table 2. We did not find significant results for sex specific analysis for men and women (Table S2). Figure 2 summarizes subgroup analysis in all study participants. We found significant interaction between coffee consumption and age. Thus, the subgroup analysis found that more than two servings of daily coffee intake were significantly associated with hypertension (OR, 0.76; 95% CI, 0.65–0.89) in group where age was greater than median. In adults who had normal cholesterol concentration more than two servings of daily coffee intake (OR, 0.84; 95% CI, 0.70–0.99) was significantly inversely associated with hypertension (Fig. 2).

**Table 2:** Logistic regression analysis of association of daily coffee consumption with hypertension

Model	Adjusted odds ratio (95% confidential interval)	
	≤ 2 Servings per day	> 2 Servings per day
Model 1^a)^	1 (Reference)	0.85 (0.74–0.97)^*^
Model 2^b)^	1 (Reference)	0.84 (0.72–0.98)^*^
Model 3^c)^	1 (Reference)	0.84 (0.73–0.99)^*^
Propensity score-matched analysis^d)^	1 (Reference)	0.83 (0.69–0.98)^*^

^a)^Adjusted for age and sex; ^b)^Model 1 + education, body mass index, current smoking, heavy drinking, diabetes, and hypercholesterolemia; ^c)^Model 2 + energy intake, income, and area of residence; ^d)^Adjusted for age, sex, education, body mass index, current smoking, heavy drinking, diabetes, hypercholesterolemia, energy intake, income, and area of residence in propensity score-matched data. ^*^P < 0.05

## Discussion

In the present study, we evaluated the association of coffee consumption and hypertension using the KNHANES data. We found that individuals consuming > 2 servings of coffee in a day were associated with reduced odds for hypertension. Participants having age greater than media value had significantly inverse association with hypertension.

A study by Miranda et al. [6] found that moderate coffee intake had beneficial effect on the risk of hypertension only in nonsmokers. Another study found inverse association between regular coffee consumption and risk of hypertension in women [10]. A polish cohort study found that consumption of four servings of coffee per day decreased the risk of hypertension [19]. While two meta-analysis of cohort studies provided quantitative evidence of coffee consumption and inverse association with hypertension [20, 21]. Another Asian cohort found association of more than three cups of coffee intake daily and reduced risk of hypertension [22]. A recent review published that moderate and habitual coffee consumption does not affect arterial BP [23]. Habitual coffee consumption was not associated with an increase in BP [24]. Coffee drinking increased BP in non-habitual drinkers but not in habitual coffee drinkers. Our results are in agreement with these previous studies and meta-analysis.

Coffee’s major ingredient is caffeine [25]. Caffeine has an acute pressor effect which is regulated by adenosine receptor, activation of sympathetic nervous system via elevating catechol amines and stimulating pitutary-adreno cortisol response, and increase cortisol production [26]. However, habitual coffee drinker develops tolerance to the caffeine-induced pressor effect [27]. A complex set of counter regulatory hormones, that maintain BP, may have caused tolerance to the humoral and hemodynamic effects of caffeine [28]. Another compound, cholinergic acid exhibits anti-inflammatory properties by inhibiting the activity of angiotensin converting enzyme through reduced production of NAD(P)H-dependent superoxide. Coffee also contains soluble fiber, polyphenols, and potassium, which may have beneficial effects on BP [13]. These ingredients may counterbalance caffeine’s pressor effect above a certain level of consumption [29]. This may help to explain the inverse relationship between habitual coffee intake and hypertension in our study. Furthermore, individual sensitivity to genetic factors like CYP1A2 and ND2-237 can affect the action of coffee on BP [30, 31]. Subgroup analysis in Fig. 2 showed significant interaction of coffee intake and age, as coffee consumption may differ by age. A study in Korean population found an increase in coffee consumption in age group 40 to 59 years in comparison to other age groups [7]. Our results are interpretable with the previous findings, as our study age group of age greater than median is comparable. We did not find significant results for association of daily coffee consumption with hypertension by smoking status or sex, it could have been due to multiple comparison between subgroups. Studies by Palatini et al. [32], Hu et al. [33], and Uiterwaal et al. [34] did not find any differences according to sex or smoking status of participants. Coffee is consumed in many forms such as brewed coffee, boiled coffee, and instant coffee mix. Instant coffee consumption has found to be associated with increased waist circumference and lipid profiles [35, 36]. However, we were unable to use types of coffee in our research, as the FFQ data did not have the information on type of coffee consumed.

Our studies have some limitations. First, due to cross-sectional nature of our study, causal associations between coffee consumption and hypertension could not be confirmed. Second, the target group of the study is young, thus we conducted propensity score analysis to minimize the influence of unbalanced characteristic of age. Third, there could be a possibility of recall bias for the frequency of coffee consumption filled by FFQ. Fourth, we did not include the amounts of caffeine, added sugar, creamer in the statistical model, due to the limitation of KNHANES data. Last, there was potential for unmeasured and residual confounding factors, which are a common problem in observational studies.

Our study had several strengths too. First, to the best of our knowledge, the present study is the first study in Korean population to demonstrate the association of coffee consumption and hypertension in adults. Second, well-defined analysis was performed, and availability of a wide range of lifestyle and dietary factors allowed to adjust for multiple confounders. Thereby, recommending further studies to strongly assess the biochemical and behavioral characteristics in each subgroup.

## Conclusions

In conclusion, the results from this study indicate that > 2 servings of coffee consumption per day had an inverse association with hypertension in Korean adults compared with ≤ 2 servings per day. Larger size cohort with longer follow up studies are required to focus on the causality of the relationship between coffee intake and hypertension.

## Electronic supplementary material

Below is the link to the electronic supplementary material.

Supplementary Material 1

## Data Availability

Not applicable.

## List of Abbreviations

BMI: Body mass index.
BP: Blood pressure.
CI: Confidence interval.
ESH: European Society of Hypertension.
FFQ: Food frequency questionnaire.
KDCA: Korea Disease Control and Prevention Agency.
KNHANES: Korean National Health and Nutrition Examination Survey.
OR: Odds ratio.
SQFFQ: Semi-quantitative food frequency questionnaires.
