# Supplementary material for: Coffee intake and hypertension in Korean adults: results from KNHANES 2012–2016
Source: Clin Hypertens. 2023 Jul 15;29:20. doi: 10.1186/s40885-023-00239-4 (PMC10349480; doi:10.1186/s40885-023-00239-4)
Supplement: Supplementary file 1 — Supplementary Material 1 [file 40885_2023_239_MOESM1_ESM.docx]

**Table S1.** Distribution of coffee intake and hypertension by sex

| Variable | Total study population (n = 12,133) | | | After propensity score matching (n = 5,172) | | |
| --- | --- | --- | --- | --- | --- | --- |
|  | Men  (n = 5,303) | Women  (n = 6,830) | P-value | Men  (n = 2,987) | Women  (n = 2,185) | Standardized mean difference |
| Hypertension |  |  | 0.02 |  |  | –0.01 |
| No | 3,900 (77) | 5,874 (86) |  | 2,146 (72) | 1,858 (85) |  |
| Yes | 1,403 (23) | 956 (14) |  | 841 (28) | 327 (15) |  |
| Coffee intake |  |  | <0.01 |  |  | 0.01 |
| ≤2 Cups | 3,592 (68) | 5,668 (83) |  | 1,494 (50) | 1,092 (50) |  |
| >2 Cups | 1,711 (32) | 1,162 (17) |  | 1,493 (50) | 1,093 (50) |  |

Data are presented as number (%).

**Table S2.** Logistic regression analysis of association of daily coffee consumption with hypertension by sex

| Daily coffee consumption | Adjusted odds ratio (95% confidential interval) | | | |
| --- | --- | --- | --- | --- |
|  | Model 1^a)^ | Model 2^b)^ | Model 3^c)^ | Propensity score-matched analysis^d)^ |
| Man |  |  |  |  |
| ≤2 Servings (n = 3,592) | 1 (Reference) | 1 (Reference) | 1 (Reference) | 1 (Reference) |
| >2 Servings (n = 1,711) | 0.90 (0.77–1.05) | 0.88 (0.74–1.06) | 0.88 (0.75–1.07) | 0.86 (0.69–1.05) |
| Woman |  |  |  |  |
| ≤2 Servings (n = 5,668) | 1 (Reference) | 1 (Reference) | 1 (Reference) | 1 (Reference) |
| >2 Servings (n = 1,162) | 0.83 (0.69–1.06) | 0.82 (0.64–1.04) | 0.82 (0.65–1.05) | 0.81 (0.60–1.07) |

^a)^Adjusted for age and sex; ^b)^Model 1 + education, body mass index, current smoking, heavy drinking, diabetes, and hypercholesterolemia; ^c)^Model 2 + energy intake, income, and area of residence; ^d)^Adjusted for age, sex, education, body mass index, current smoking, heavy drinking, diabetes, hypercholesterolemia, energy intake, income, and area of residence in propensity score-matched data.
